# Do Autophagy Enhancers/ROS Scavengers Alleviate Consequences of Mild Mitochondrial Dysfunction Induced in Neuronal-Derived Cells?

**DOI:** 10.3390/ijms22115753

**Published:** 2021-05-27

**Authors:** Damri Odeya, Natour Sarya, Agam Galila

**Affiliations:** Mental Health Center, Pharmacology and Psychiatry Research Unit, Department of Clinical Biochemistry, Faculty of Health Sciences, Ben-Gurion University of the Negev, Beer-Sheva 8461144, Israel; odeyad@post.bgu.ac.il (D.O.); sarya@post.bgu.ac.il (N.S.)

**Keywords:** rotenone, mitochondrial dysfunction, autophagy enhancers, ROS scavengers, bipolar disorder

## Abstract

Mitochondrial function is at the nexus of pathways regulating synaptic-plasticity and cellular resilience. The involvement of brain mitochondrial dysfunction along with increased reactive oxygen species (ROS) levels, accumulating mtDNA mutations, and attenuated autophagy is implicated in psychiatric and neurodegenerative diseases. We have previously modeled mild mitochondrial dysfunction assumed to occur in bipolar disorder (BPD) using exposure of human neuronal cells (SH-SY5Y) to rotenone (an inhibitor of mitochondrial-respiration complex-I) for 72 and 96 h, which exhibited up- and down-regulation of mitochondrial respiration, respectively. In this study, we aimed to find out whether autophagy enhancers (lithium, trehalose, rapamycin, and resveratrol) and/or ROS scavengers [resveratrol, *N*-acetylcysteine (NAC), and Mn-Tbap) can ameliorate neuronal mild mitochondrial dysfunction. Only lithium (added for the last 24/48 h of the exposure to rotenone for 72/96 h, respectively) counteracted the effect of rotenone on most of the mitochondrial respiration parameters (measured as oxygen consumption rate (OCR)). Rapamycin, resveratrol, NAC, and Mn-Tbap counteracted most of rotenone’s effects on OCR parameters after 72 h, possibly via different mechanisms, which are not necessarily related to their ROS scavenging and/or autophagy enhancement effects. The effect of lithium reversing rotenone’s effect on OCR parameters is compatible with lithium’s known positive effects on mitochondrial function and is possibly mediated via its effect on autophagy. By-and-large it may be summarized that some autophagy enhancers/ROS scavengers alleviate some rotenone-induced mild mitochondrial changes in SH-SY5Y cells.

## 1. Introduction

Mitochondria are most widely known as the power workshop of the cell, due to their production of adenosine triphosphate (ATP) through the electron transport chain and oxidative phosphorylation (OXPHOS) pathways [1]. Besides their role in energy production, mitochondria play crucial roles in a myriad of cellular functions, including differentiation, cell cycle, cell growth, intracellular Ca^+2^ buffering and generation of reactive oxygen species (ROS) [2]. Hence, mitochondria are also involved in apoptosis [3,4,5,6,7] and autophagy [1,6,8,9,10,11]. Mitophagy (autophagy of mitochondria) is the organelle’s quality control pathway which helps cells to selectively eliminate and recycle damaged and uncoupled mitochondria that are beyond repair, while preserving healthy mitochondria functioning above a certain threshold, consequently preventing further damage leading to cell death. ROS scavengers also directly correct mitochondrial dysfunction by decreasing intracellular Ca^2+^ levels and modifying mitochondrial Ca^2+^ dynamics [12], and inhibit apoptotic pathways [13].

Neuronal cells have a high energy demand. While the brain constitutes only about 2% of the body’s mass, it consumes, on average, 20% of the body’s total energy [14]. This makes neuronal cells almost entirely dependent on glucose oxidation for ATP production [15]. Thus, a proper functional mitochondrial network with sufficient and persistent ATP production is essential for neuronal survival and function. Additionally, highly dynamic calcium fluctuations happen inside neurons, requiring their steady buffering capacity [16]. It has recently been suggested that aberrant neuronal function caused by mitochondrial dysfunction may be ameliorated either by ROS scavenging or by enhancing mitophagy [17,18,19,20,21]. Due to the postmitotic nature and metabolic dependence on mitochondria, insufficient or dysregulated mitophagy is detrimental to neurons. Indeed, Defects in mitophagy have been implicated in neuropsychiatric disorders [18,22].

Here we recapitulated our recently reported model of mild mitochondrial dysfunction [23] in the human neuron-derived cell line, SH-SY5Y, induced by low rotenone (a mitochondrial complex I inhibitor) concentrations never used before, to examine whether autophagy enhancers or ROS scavengers which are either generally accepted as safe (GRAS) compounds or approved drugs (mood stabilizers) can amend neuronal mild mitochondrial dysfunction. The rationale for using ROS scavengers was that higher rotenone doses, regularly used to model Parkinson’s disease (PD) [24], have been reported to raise mitochondrial ROS production, a crucial player in apoptosis [25]. The rationale for using autophagy/mitophagy enhancers was (a) assuming that degradation of dysfunctional mitochondria may counteract rotenone-induced changes; (b) rotenone treatment of primary neurons has been reported to induce redistribution of cardiolipin (CL) from the inner to the outer mitochondrial membrane. LC3 was found to bind to CL; preventing this interaction inhibited rotenone-induced delivery of mitochondria to autophagosomes and lysosomes and attenuated mitophagy [26,27]. The rationale for using GRAS agents was that if found to contribute to neuronal wellbeing, they could relatively quickly be implemented as novel drugs for central nervous system (CNS) disorders caused by mitochondrial malfunction.

Previous studies have used other approaches/types of molecules to rescue mitochondrial function. For example, based on its multiple mechanisms of action, ursodeoxycholic acid (UDCA), utilized in the treatment of liver diseases, was studied in the rotenone-induced PD model in rats [28]. Kim et al. used the paradigm of glutamate-induced oxidative stress and neuronal cell death in HT-22 cells to study the mechanism of the protective effects of Ole, the polyphenolic compound oleuropein [29]. They report that exposure to glutamate caused neuronal cell death through an alteration of Bcl-2-like protein 4 (Bax)/B-cell lymphoma (Bcl)-2 expression and translocation of mitochondrial apoptosis-inducing factor (AIF) to the cytoplasm of the cells, induced an increase in dephosphorylation of dynamin-related protein 1 (Drp1), mitochondrial fragmentation, and mitochondrial dysfunction. Pretreatment with Ole decreased Bax expression, increased Bcl-2 expression, inhibited the translocation of mitochondrial AIF to the cytoplasm, amended mitochondrial dynamic imbalance, reduced the number of cells with fragmented mitochondria, and regulated the phosphorylation of Drp1, a GTPase that regulates mitochondrial fission.

In the present study, SH-SY5Y cells were exposed to rotenone only, the pharmaceuticals (autophagy enhancers or ROS scavengers) only, or to the pharmaceuticals following the incubation with rotenone. Manifold mitochondrial function-related parameters were assessed: cell viability, mitochondrial respiration, ATP levels, and autophagy markers. Each of the pharmaceuticals differently affected the parameters tested regardless of their assumed mechanism (autophagy enhancement or ROS scavenging).

## 2. Materials and Methods

### 2.1. Cell Culture

Human neuroblastoma cells, SH-SY5Y (ATCC, Manassas, VA, USA), were maintained in DMEM medium (Biological Industries, Beit Haemek, Israel) supplemented with 1% fetal bovine serum (FBS, Biological Industries, Beit Haemek, Israel) at 37 °C with 5% CO_2_ and 95% O_2_ (*v/v*). The cells were plated at a density of 1 × 10^4^ cells/well in 96-well plates. The cultures were grown for 24 h after which the medium was changed to containing either 10 pM of rotenone dissolved in DMSO (Sigma-Aldrich, St. Louis, MO, USA) or vehicle for 72, or 96 h to induce mitochondrial dysfunction. Drugs were delivered after 48 h of 10 pM rotenone. Cells were treated with either of the autophagy enhancers trehalose (50 mM, obtained from The Endowment for Medical Research, Huston, TX) dissolved in water, rapamycin (10 nM, Sigma-Aldrich, St. Louis, MO.) dissolved in DMSO, lithium (1 mM, Sigma-Aldrich, ibid) dissolved in water and resveratrol (50 µM, Sigma-Aldrich, ibid) dissolved in DMSO for 24/48 h or the ROS scavengers N-acetylcysteine (NAC, 100 nM, Sigma-Aldrich, ibid) dissolved in water and Mn(III)tetrakis (4-benzoic acid) porphyrin (Mn-Tbap, 3 µM, Sigma-Aldrich, ibid) dissolved in DMSO.

### 2.2. Determination of Cell Viability by the MTT Assay

Cell viability (based on mitochondrial succinate dehydrogenase activity) was assessed by the quantitative colorimetric eukaryotic cell survival assay based on MTT (3-(4,5-dimethylthiazol-2-yl)-2,5-diphenyltetrazolium bromide, Sigma, ibid) dissolved in Dulbecco’s Phosphate-Buffered Saline (DPBS) without calcium and magnesium (Biological Industries, ibid). The assay detects living, but not dead, cells. Briefly, MTT stock solution (2.5 mg/mL, 20% in the culture medium) was added to the cell culture at 37 °C for 2 h. An equal volume of DMSO was then added, and the cell culture was placed on a shaking table for about two minutes until the resultant formazan crystals dissolved. Absorbance was read at 540 nm, and background absorbance—at 690 nm (ELISA reader, Labsystems, Helsinki, Finland). Wells without cells were used as ‘blanks’ and were subtracted as background from each sample. Results are expressed as a percent of control. The MTT assay is based on MTT reduction by mitochondrial succinate dehydrogenase, and thus, also provides information concerning mitochondrial respiration [30].

### 2.3. Determination of Mitochondrial Respiration Parameters

Oxygen Consumption Rate (OCR) was measured using the Seahorse XF-24 Extracellular Flux Analyzer (Seahorse Biosciences, North Billerica, MA, USA). On the day prior to the experiment, the cells were seeded, 8 × 10^3^ cells/well for 96 h experiments, to 50 × 10^3^ cells/well for acute experiments, in 600 μL growth media in XF plates. An hour prior to the assay, the medium was changed to DMEM without sodium bicarbonate (Biological Industries, ibid), and cells were incubated in CO_2_-free environment for 60 min at 37 °C to reach temperature and pH equilibration. Thereafter, the plate was inserted into the Seahorse Analyzer for two hours. The apparatus monitors OCR every 10 min., first with no injection for 30 min. followed by consecutive injections of three mitochondrial respiration inhibitors every 30 min.: (i) Oligomycin (1.5 µM), an ATP-synthase (complex V) blocker; (ii) FCCP (1 μM) [Carbonyl cyanide 4-(trifluoromethoxy) phenylhydrazone], an uncoupling agent that collapses the proton gradient and disrupts mitochondrial membrane potential; (iii) rotenone (1 μM), mitochondrial ETC’s complex I inhibitor (all from Sigma-Aldrich, ibid). Each sample was assayed in triplicate. Basal OCR, proton leak, maximal respiration, spare capacity, nonmitochondrial oxygen consumption, and ATP-linked OCR were all calculated using Seahorse Bioscience software (version 3.3). Results were then normalized to protein concentration in each well.

### 2.4. Determination of Protein Concentration

Total protein was extracted from the cells after their harvest from the plate. Extraction was carried out by sonication for 10 s at 4 °C and 50% power capacity (Heat System Ultrasonic, Newtown, CT, USA) in 100 µL RIPA lysis buffer [50 mM Tris HCl pH 7.5, 150 mM NaCl, 1 mM ethylenediaminetetraacetic acid (EDTA), 1% NP-40, 1% sodium deoxycholic acid, 0.1% sodium dodecyl sulfate (SDS), and the following ingredients added freshly—1 mM phenylmethylsulfonyl fluoride (PMSF), 1 µL proteases inhibitor cocktail, 1 µL phosphatase inhibitor cocktail (all from Sigma-Aldrich, ibid). After centrifugation at 10,000× *g* for 15 min at 4 °C, the supernatant was collected, and protein concentration determined spectrophotometricaly at 280 nm using NanoDrop 2000 (Thermo Scientific, Waltham, MA, USA).

### 2.5. Determination of ATP Levels

ATP in lyzed cells was measured using a colorimetric kit (Abcam, Cambridge Science Park, Cambridge, UK), generating a quantifiable glycerol phosphorylation product according to the manufacturer’s recommendations. The absorbance of the samples is read at 540 nm, and background absorbance—at 690 nm (ELISA reader, Labsystems, Helsinki, Finland).

### 2.6. Determination of Mitochondrial OXPHOS Complex I Enzymatic Activity

Complex I enzymatic activity was measured using a dedicated kit (ab109721, Abcam, ibid). Absorbance was read at 450 nm (ELISA reader, Labsystems, ibid). Wells without cells were used as background subtracted from the result of each well.

### 2.7. Western Blotting

Western blotting was performed according to a standard protocol used in our laboratory [31] on 10% acrylamide gel and transferred to PVDF membrane. Each sample was tested in duplicates of 10 and 20 µg/lane, to verify linearity. Primary antibodies and their dilutions in TBST (A mixture of tris-buffered saline (TBS) and Tween 20) were: Total OXPHOS cocktail (for CoI—subunit NDUFB8, for CoII—SDHB, for CoIII—Core protein 2, for CoIV—subunit I, and for CoV—alpha subunit; 1:1500, Abcam, ibid), p62 (1:1500, Abcam, ibid) and LC3 (1:1000, Sigma-Aldrich, ibid). Secondary rabbit antibodies (sc2004, 1:10,000, Santa Cruz Biotechnology, Dallas, TX, USA) were also diluted in TBST. Results were normalized to Ponceau staining (total protein [32], Sigma-Aldrich, ibid).

### 2.8. Statistical Analysis

Results are given as means ± SEM either of the original values or of the % of the mean of the control. The latter normalization was carried out for experiments preformed several times at different time points and/or using different batches of kits/plates. Results exceeding +/−2SDs were excluded. Statistical analysis was carried out by two-way ANOVA followed by Fisher’s Least Significant Difference (LSD) post-hoc test, using STATISTICA version 13 (StatSoft, Tulsa, OK, USA). *p* ≤ 0.05 was considered statistically significant.

Data from the Seahorse XF Apparatus were exported to Excel 2010 for further analysis. The serial nature of the measurements obtained using the apparatus’ software allowed repeated-measures ANOVA for some of the experiments.

## 3. Results

We have recently modeled very mild mitochondrial dysfunction by exposing SH-SY5Y cells to very low rotenone doses in vitro and by chronically injecting (in vivo) mice with very low rotenone doses [23]. In the present study, we examined in the SH-SY5Y cells (in vitro) whether any of the autophagy enhancers trehalose [33], rapamycin [34], and lithium [35], or the ROS scavengers NAC [36] and Mn-Tbap [37], or resveratrol, both an autophagy enhancer and a ROS scavenger [38], reverse the consequences of mild mitochondrial dysfunction. The paradigm was constructed to mimic the situation in real life. Namely, first, the damage occurs, and thereafter, the remedy is sought. Thus, the cells were first exposed to rotenone for 72 or 96 h, and the tested compounds were added for the last 24/48 h, respectively, of the exposure to rotenone. As an inevitable control, we evaluated the effect of exposure to the autophagy enhancers and the ROS scavengers for 24 and 48 h on a myriad of mitochondrial function-related parameters (Table 1). We hereby relate only to results that reached statistical significance: A. autophagy enhancers—*lithium* (1 mM) for 24 h elevated the levels of the autophagy markers LC3-II; exposure to lithium for 48 h also reduced mitochondrial basal OCR. *Trehalose* (50 mM) for 24 h elevated nonmitochondrial OCR, ATP levels, and CoIV protein levels; 48 h of trehalose led to elevated ATP levels. 24 h of exposure to *rapamycin* (10 nM) resulted in elevated mitochondrial maximal respiration and spare respiratory capacity; 48 h of rapamycin resulted in reduced mitochondrial proton leak and nonmitochondrial respiration, but elevated ATP levels. B. The autophagy enhancer and ROS scavenger *Resveratrol* (50 µM) reduced cell viability (MTT assay) and ATP levels following 24 h of exposure; exposure to resveratrol for 48 h led to elevated levels of cell viability (multiplex assay), mitochondrial mass, ATP levels and CoIII protein levels and to reduced levels of mitochondrial basal OCR, maximal respiration, spare respiratory capacity and ATP-linked OCR. C. ROS scavengers—*NAC* (100 nM) affected the parameters only following 24 h of exposure. It increased ATP levels. *Mn-Tbap* (3 µM)—exposure for 24 h elevated CoIV protein levels but reduced mitochondrial mass and mitochondrial basal OCR and proton leak. Mn-Tbap added for 48 h increased cell viability (MTT assay), non-mitochondrial respiration, and ATP levels, while reducing mitochondrial basal OCR, proton leak, maximal respiration, spare respiratory capacity and ATP-linked OCR.

**Figure 1:**
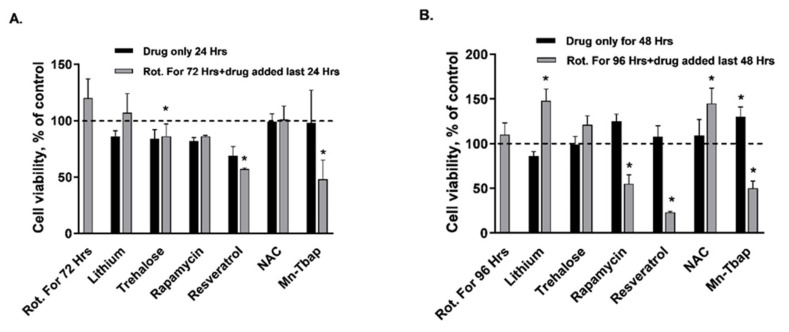
Effect of ROS scavengers/autophagy enhancers on cell viability pre and post exposure to 10 pM rotenone.

For the reversal studies the cells were exposed to 10 pM rotenone for either 72 or 96 h, and the drugs (same concentrations as above) were added for the last 24/48 h, respectively. All the observed changes are summarized in Table 2. Detailed statistical analyses of the data described below are given in the figure legends. Rotenone treatment did not affect cell viability (MTT assay), but there was an interaction between the effect of the autophagy enhancers and the ROS scavengers with that of rotenone on cell viability in a duration-dependent manner (Figure 1A,B). Although lithium and *NAC* by themselves did not affect cell viability, their addition for the last 48 h of the exposure to rotenone for 96 h significantly increased this parameter beyond the values obtained with rotenone only, as well as with those of the untreated cells (control) (Figure 1A,B). *Trehalose*, *resveratrol*, and *Mn-Tbap,* each added for the last 24 h of the exposure to rotenone for 72 h, significantly reduced cell viability compared to the values following exposure to rotenone only and as compared to the control (Figure 1A,B). In a similar manner, when added for the last 48 h of the exposure to rotenone for 96 h, each of *rapamycin*, *resveratrol* and *Mn-Tbap* significantly decreased cell viability as compared with cells exposed to rotenone only, to control cells and to cells exposed to the drugs themselves (Figure 1A,B). Neither the autophagy enhancers nor the ROS scavengers as a group demonstrated a consistent effect, suggesting that the mechanisms by which these drugs affect cell viability are not mediated by their autophagy enhancing/ROS scavenging properties.

Results are means + S.E.M. of three experiments each in triplicate, expressed as percent of control. Control values were 0.336 ± 0.034 OD_540 nm_. Rot. = rotenone; Li = lithium; Tre. = trehalose; Rap. = rapamycin; Res. = resveratrol; NAC = N-acetylcysteine; Mn. = Mn-Tbap. **A**. Cell viability following exposure to rotenone for 72 h. and to ROS scavengers/autophagy enhancers during the last 24 h. of rotenone. ANOVA, F_13,42_ = 5.04, *p* = 0.00001; Fisher’s LSD post-hoc test: * Rot. + Tre., Res., Rot. + Res. and Rot. + Mn-Tbap vs. control and vs. Rot. for 72 h, *p* ≤ 0.04. **B**. Cell viability following exposure to rotenone for 96 h. and to ROS scavengers/autophagy enhancers during the last 48 h. of rotenone. ANOVA, F_13,42_ = 11.4, *p* = 0.000001; Fisher’s LSD post-hoc test: * Rot. + Li, Rot. + Rap., Rot. + Res., Mn-Tbap and Rot. + Mn-Tbap vs. control and vs. Rot. For 96 h, *p* ≤ 0.02.

**Figure 2:**
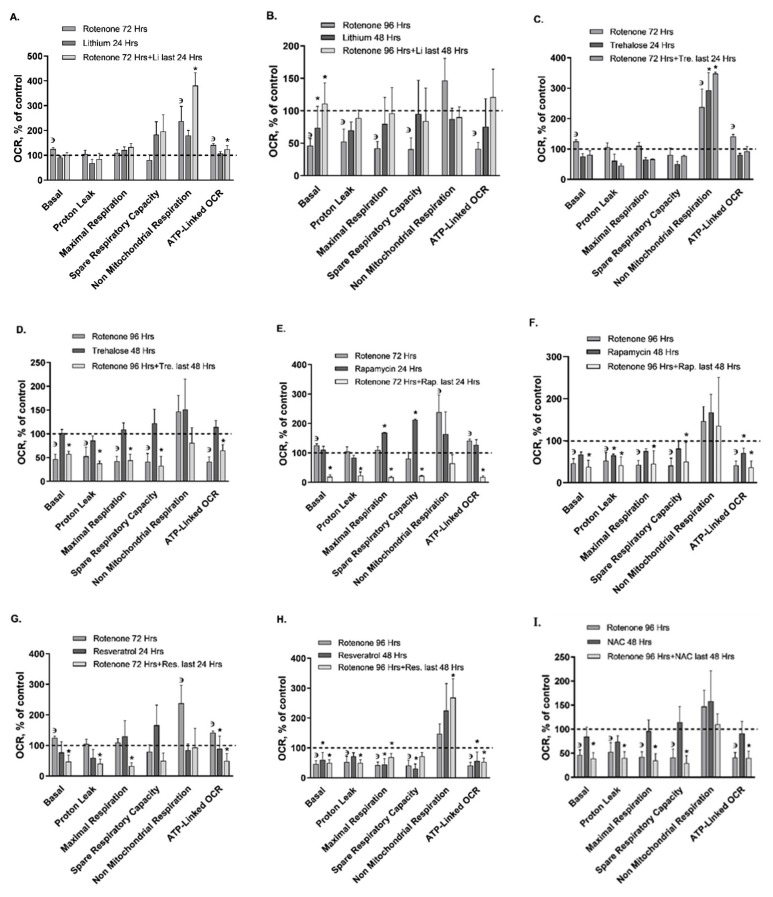

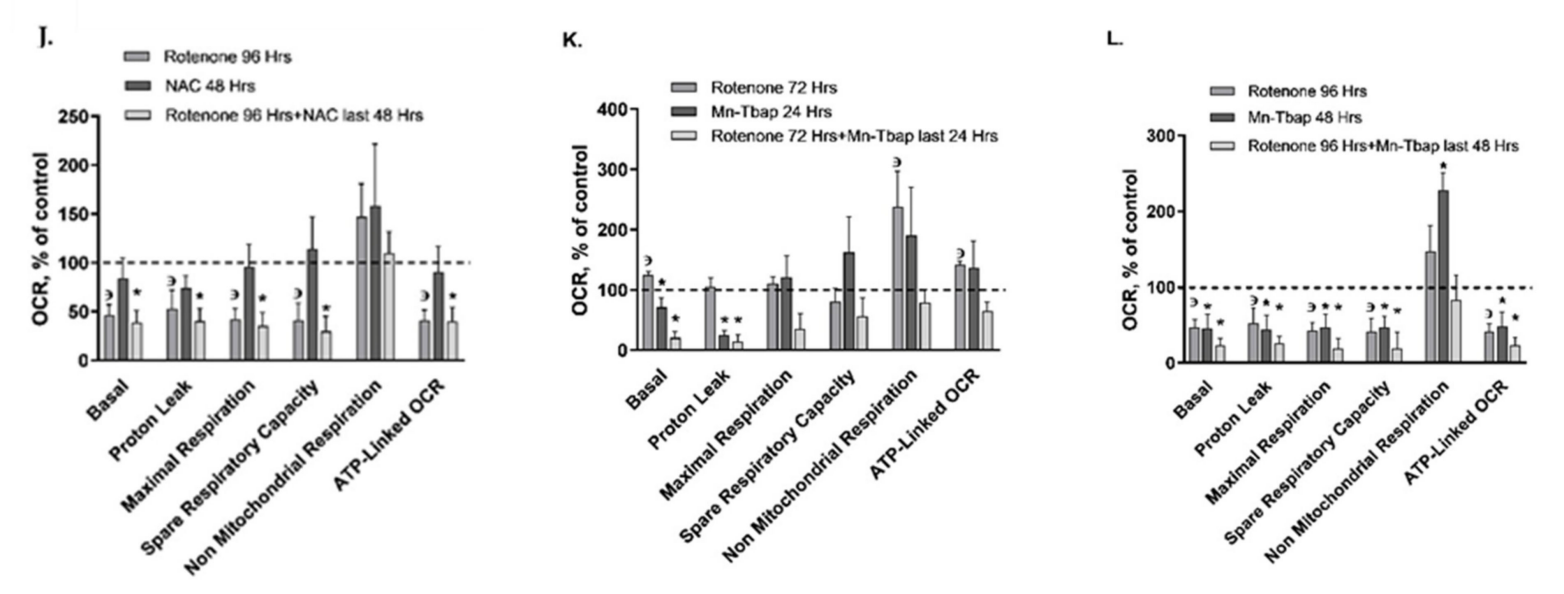
Effect of ROS scavengers/autophagy enhancers on mitochondrial respiration parameters pre and post exposure to 10 pM rotenone for 72 (**A,C,E,G,I,K**) or for 96 (**B,D,F,H,J,L**) h.

As we reported earlier [23], the predominant effect of 10 pM, rotenone for 72 and 96 h on SH-SY5Y cells was on mitochondrial respiration parameters. We, therefore, now assessed the effect of the autophagy enhancers and the ROS scavengers added for the last 24/48 h of the exposure to rotenone for 72/96 h, respectively, on mitochondrial and nonmitochondrial respiration (OCR parameters) (Figure 2; Table 2). A counteracting effect of a drug on rotenone’s effect is concluded when the effect of the drug added for the last 24/48 h of the exposure to rotenone is statistically significant from that of rotenone. An intuitive counteraction is deduced when the effect of the drug added for the last 24/48 h of the exposure to rotenone is statistically nonsignificant from the control, from which rotenone only is significantly different. *Lithium* added for the last 24 h of the 72 h of the exposure to rotenone reversed rotenone’s effect on basal mitochondrial respiration (Figure 2A; Supplementary Figure S1A; Table 2). Lithium added for the last 48 h of the 96 h of the exposure to rotenone reversed rotenone’s effects on all mitochondrial respiration parameters (Figure 2B; Supplementary Figure S1B; Table 2). This result is compatible with lithium’s known enhancing effect on mitochondrial OXPHOS [35,39,40]. *Trehalose* added for the last 24 h of the 72 h of the exposure to rotenone reversed rotenone’s effects on basal mitochondrial respiration and ATP-linked OCR (Figure 2C; Table 2). The autophagy enhancer *rapamycin, resveratrol* (both an autophagy enhancer and a ROS scavenger), and the ROS scavengers *NAC* and *Mn-Tbap*, each added for the last 24 h of the exposure to rotenone for 72 h, counteracted all rotenone-enhanced OCR parameters (Figure 2E,G,I,K; Table 2). Trehalose, rapamycin, resveratrol, NAC, and Mn-Tbap added for the last 48 h of the exposure to rotenone for 96 h did not counteract rotenone’s effects (Figure 2D,F,H,J,L; Table 2).

**Figure 3:**
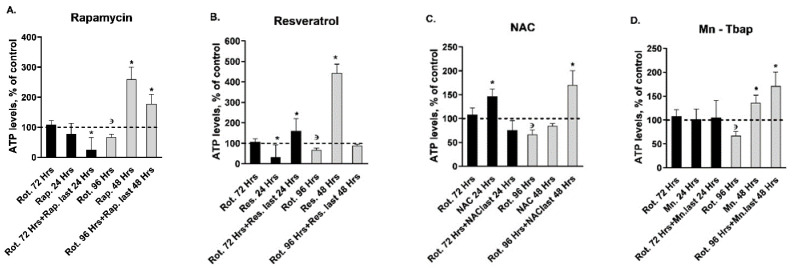
Effect of ROS scavengers/autophagy enhancers on ATP Levels pre and post exposure to 10 pM rotenone.

Given the variability in the response/non-response of the mitochondrial respiration parameters to the different autophagy enhancers and ROS scavengers, we further studied these drugs’ effects on adenosine triphosphate (ATP) levels, CoI activity, and mitochondrial respiration complexes’ protein levels—all parameters related to rotenone’s effects per se. Although intracellular ATP levels were significantly altered (decreased) only following exposure to rotenone for 96 h (Figure 3), *rapamycin and resveratrol*, when added for the last 24 h of the exposure to rotenone for 72 h, significantly affected ATP levels in opposite directions. While rotenone+rapamycin significantly reduced the levels, rotenone+resveratrol increased them (Figure 3A,B; Table 2). *Rapamycin, resveratrol, NAC, and Mn-Tbap* added for the last 48 h of the exposure to rotenone for 96 h significantly counteracted rotenone’s effect (Figure 3A–D; Table 2). *Lithium and trehalose* did not affect rotenone’s effects on ATP levels (Table 2). As in the case of cell viability, neither the autophagy enhancers nor the ROS scavengers as a group exhibited a consistent effect, supporting the notion that the mechanisms by which these drugs affect ATP levels are also, apparently, not mediated by their autophagy enhancing/ROS scavenging properties.

As already recently described by Damri et al. [23], among the five mitochondrial respiration complexes, rotenone significantly affected only CoI and CoIV protein levels. Specifically, exposure to rotenone significantly enhanced/decreased CoI activity following 72/96 h, respectively. All drugs added for the last 24/48 h of the exposure to rotenone for 72/96 h, respectively, counteracted rotenone’s effect (Table 2). Noticeably, *rapamycin* added for the last 24 h of the exposure to rotenone for 72 h not only normalized rotenone-induced increase. It significantly decreased CoI protein levels as compared to control levels (Table 2). As for CoIV, the increase in its levels following 72 h of rotenone was counteracted by all drugs tested except for Mn-Tbap, which further significantly augmented the increase (Table 2). The increase in CoIV levels following 96 h of rotenone was counteracted by all drugs tested except for trehalose (Table 2). Here, again, the effects of the drugs do not conform to common outcomes of autophagy enhancers or of ROS scavengers.

**Figure 4:**
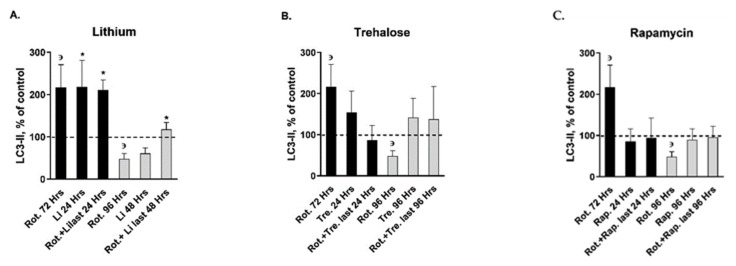

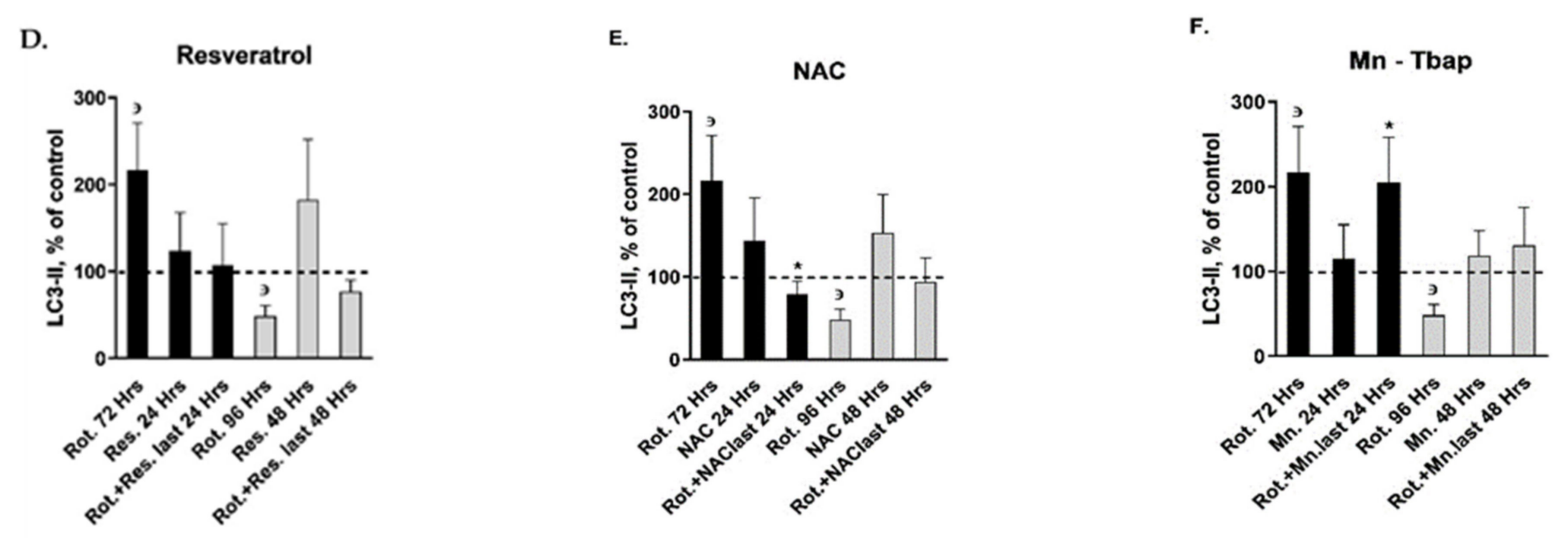
Effect of autophagy enhancers/ROS scavengers on LC3-II protein levels pre and post exposure to 10 pM rotenone Results represent means ± S.E.M. of four independent experiments, each in duplicate. Rot. = rotenone; Li = lithium; Tre. = trehalose; Rap. = rapamycin; Res. = resveratrol; NAC = N-acetylcysteine; Mn. = Mn-Tbap. ^з^Rot. for 72/96 h vs. control, *p* = 0.05—as previously described [23] and confirmed now. One sample of each of rotenone’s effect, NAC’s effect and Mn-Tbap’s effect for 72 h exceeding mean ± 2SD was omitted. **A.** Lithium effects, Two-way ANOVA: Treatment—F_3,25_ = 3.02, *p* = 0.04; duration—F_1,25_ = 42.7, *p* = 0.00001; TreatmentXDuration interaction—F_3,25_ = 7.6, *p* = 0.0008; *Fisher’s LSD post-hoc test: 24 h of Li vs. control, *p* < 0.00002; Rot. for 72 h + Li. last 24 h vs. control, *p* = 0.008; Rot. for 96 h + Li. last 48 h vs. Rot. for 96 h, *p* = 0.05. **B.** Trehalose effects, Two-way ANOVA: TreatmentXDuration interaction—F_3,26_ = 2.5, *p* = 0.07. **C.** Rapamycin effects, Two-way ANOVA: TreatmentXDuration interaction—F_3,26_ = 4.2, *p* = 0.01. **D.** Resveratrol effects, Two-way ANOVA: TreatmentXDuration interaction—F_3,24_ = 3.2, *p* = 0.03. **E.** NAC effects, Two-way ANOVA: TreatmentXDuration interaction—F_3,26_ = 3.8, p = 0.02; *Fisher’s LSD post-hoc test: 72 h of Rot. + NAC last 24 h vs. 72 h of Rot, *p* < 0.005. **F.** Mn-Tbap effects, Two-way ANOVA: Duration—F_1,25_ = 4.7, *p* = 0.03; TreatmentXDuration interaction—F_3,25_ = 3.9, *p* = 0.01; *Fisher’s LSD post-hoc test: 72 h of Rot.+Mn. last 24 h vs. control, *p* = 0.02.

Induction of mitochondrial distress (by rotenone in our case) is expected to enhance autophagy (mitophagy). We, therefore, assessed the levels of the two autophagy markers LC3-II and p62. Exposure of SH-SY5Y cells to 10 pM rotenone for 72/96 h significantly increased/decreased, respectively LC3-II protein levels (Figure 4; Table 2). All drugs tested except lithium and Mn-Tbap counteracted the effect of rotenone following 72 h of exposure, and all drugs counteracted rotenone’s effect following 96 h of exposure (Figure 4; Table 2). p62 protein levels were not affected by rotenone or rotenone + each of the drugs (Table 2).

Results (means ± S.E.M. of six experiments) were obtained in pmoles O_2_ consumed/min/mg protein and converted into % of control. Control values were: Basal OCR—8.35 ± 2.02 (S.E.M.) pmoles O_2_ consumed/min/mg protein; Proton leak—1.79 ± 0.25; Maximal respiration—18.04 ± 6.90; Spare capacity—9.70 ± 2.85; non-mitochondrial oxygen consumption—2.19 ± 1.57; ATP-linked OCR—6.55 ± 1.77. Li = lithium; Tre. = trehalose; Rap. = rapamycin; Res. = resveratrol; NAC = N-acetylcysteine. ^з^Rot. for 72 and 96 h vs. control, *p* < 0.05 as previously described [23] and confirmed now. **Lithium: A**. Added for the last 24 h of the exposure to rotenone for 72 h. One sample exceeding mean ± 2SD was omitted. Basal OCR ANOVA, F_3,13_ = 7.7, *p* = 0.003; *Fisher’s LSD post-hoc test: Rot. vs. all, *p* < 0.01. Non-mitochondrial Respiration ANOVA, F_3,13_ = 2.4, *p* = 0.04; *Fisher’s LSD post-hoc test: Rot. + Li vs. control, *p* = 0.02. ATP-Linked OCR ANOVA, F_3,13_ = 7.5, *p* = 0.003; *Fisher’s LSD post-hoc test: Rot. + Li vs. control, *p* = 0.04. **B**. Added for the last 48 h of the exposure to rotenone for 96 h. Basal OCR ANOVA, F_3,14_ = 4.6, *p* = 0.01; *Fisher’s LSD post-hoc test: Li vs. control and vs. Rot. + Li, *p* = 0.02; Rot. + Li vs. Rot., *p* = 0.01. **Trehalose: C.** Added for the last 24 h of the exposure to rotenone for 72 h. One sample exceeding mean ± 2SD was omitted. Non-mitochondrial Respiration ANOVA, F_3,14_ = 5.3, *p* = 0.01; *Fisher’s LSD post-hoc test: Tre. and Rot. + Tre. vs. control, *p* < 0.01. **D.** Added for the last 48 h of the exposure to rotenone for 96 h. Basal OCR ANOVA, F_3,14_ = 13.2, *p* = 0.0002; *Fisher’s LSD post-hoc test: Rot+Tre. vs. control and vs. Tre., *p* < 0.003. Proton Leak ANOVA, F_3,14_ = 4.2, *p* = 0.02; *Fisher’s LSD post-hoc test: Rot. + Tre. vs. control, *p* < 0.001. Maximal Respiration ANOVA, F_3,14_ = 12.1, *p* = 0.0003; Fisher’s LSD post-hoc test: *Rot. + Tre. vs. control and vs. Tre., *p* < 0.002. Spare Respiratory Capacity ANOVA, F_3,14_ = 5.4, *p* = 0.01; *Fisher’s LSD post-hoc test: Rot. + Tre. vs. control and vs. Tre., *p* < 0.01. ATP-Linked OCR ANOVA, F_3,14_ = 12.2, *p* = 0.0003; *Fisher’s LSD post-hoc test: Rot. + Tre. vs. control and vs. Tre., *p* < 0.03. **Rapamycin: E.** Added for the last 24 h of the exposure to rotenone for 72 h. Basal OCR ANOVA, F_3,13_ = 7.1, p = 0.004; *Fisher’s LSD post-hoc test: Rot.+Rap. vs. all, *p* < 0.003. Proton Leak ANOVA, F_3,14_ = 5.5, *p* = 0.01; *Fisher’s LSD post-hoc test: Rot.+Rap. vs. all, *p* < 0.02. Maximal Respiration *Fisher’s LSD post-hoc test: Rap. vs. control, *p* = 0.05; Rot.+Rap. vs. all, *p* < 0.02. Spare Respiratory Capacity ANOVA, F_3,11_ = 4.8, *p* = 0.02; *Fisher’s LSD post-hoc test: Rap. vs. all, *p* < 0.02; Rot. + Rap. vs. control, *p* = 0.04. ATP-Linked OCR ANOVA, F_3,11_ = 2.7, *p* = 0.007; *Fisher’s LSD post-hoc test: Rot. + Rap. vs. all, *p* < 0.01. **F.** Added for the last 48 h of the exposure to rotenone for 96 h. One sample exceeding mean ± 2SD was omitted. Basal OCR ANOVA, F_3,13_ = 15.4, *p* = 0.0001; *Fisher’s LSD post-hoc test: Rot. and Rot. + Rap. vs. control, *p* < 0.01. Proton Leak ANOVA, F_3,13_ = 4.5, *p* = 0.02; *Fisher’s LSD post-hoc test: Rap. and Rot. + Rap. vs. control, *p* = 0.01. Maximal Respiration ANOVA, F_3,13_ = 19.2, *p* = 0.00005; *Fisher’s LSD post-hoc test: Rap. and Rot. + Rap. vs. control, *p* < 0.05. Spare Respiratory Capacity ANOVA, F_3,13_ = 7.2, *p* = 0.004; *Fisher’s LSD post-hoc test: Rot. + Rap. vs. control and vs. Rap., *p* < 0.01. ATP-Linked OCR ANOVA, F_3,13_ = 16.1, *p* = 0.0001; *Fisher’s LSD post-hoc test: Rap. vs. all, *p* < 0.02; Rot. + Rap. vs. control and vs. Rap., *p* < 0.02. **Resveratrol: G.** Added for the last 24 h of the exposure to rotenone for 72 h. Basal OCR ANOVA, F_3,13_ = 4.8, *p* = 0.01; *Fisher’s LSD post-hoc test: Rot. + Res. vs. Rot. and vs. control, *p* < 0.04. Proton Leak ANOVA, F_3,14_ = 4.1, *p* = 0.02; *Fisher’s LSD post-hoc test: Rot. + Res. vs. control and vs. Rot., *p* < 0.01. Maximal Respiration ANOVA, F_3,11_ = 3.3, *p* = 0.05; *Fisher’s LSD post-hoc test: Rot. + Res. vs. Rot. and vs. control, *p* < 0.03. ATP-Linked OCR ANOVA, F_3,13_ = 4.7, *p* = 0.02; *Fisher’s LSD post-hoc test: Rot. + Res. vs. control, *p* < 0.002. **H.** Added for the last 48 h of the exposure to rotenone for 96 h. Basal OCR ANOVA, F_3,14_ = 6.2, *p* = 0.006; *Fisher’s LSD post-hoc test: Res. and Rot. + Res. vs. control, *p* < 0.03. Proton Leak ANOVA, F_3,14_ = 3.1, *p* = 0.04; *Fisher’s LSD post-hoc test: Rot. + Res. vs. control, *p* = 0.05. Maximal Respiration ANOVA, F_3,13_ = 8.2, *p* = 0.002; *Fisher’s LSD post-hoc test: Res. and Rot. + Res. vs. control, *p* < 0.03. Spare Respiratory Capacity ANOVA, F_3,14_ = 5.7, *p* = 0.008; *Fisher’s LSD post-hoc test: Res. vs. control, *p* < 0.003. Nonmitochondrial Respiration: ANOVA, F_3,14_ = 3.5, *p* = 0.04; *Fisher’s LSD post-hoc test: Rot. + Res. vs. Rot., *p* < 0.04. ATP-Linked OCR ANOVA, F_3,13_ = 5.6, *p* = 0.009; *Fisher’s LSD post-hoc test: Res. vs. control and Rot. + Res vs. control and vs. Res., *p* < 0.03. **NAC: I.** Added for the last 24 h of the exposure to rotenone for 72 h. Basal OCR ANOVA, F_3,14_ = 9.5, *p* = 0.0008; *Fisher’s LSD post-hoc test: Rot. + NAC. vs. all, *p* < 0.02. Proton Leak ANOVA, F_3,16_ = 8.3, *p* = 0.001; *Fisher’s LSD post-hoc test: Rot. + NAC vs. all, *p* < 0.02. ATP-Linked OCR ANOVA, F_3,15_ = 5.1, *p* = 0.01; *Fisher’s LSD post-hoc test: Rot. + NAC vs. Rot. and vs. NAC, *p* < 0.05. **J.** Added for the last 48 h of the exposure to rotenone for 96 h. Basal OCR ANOVA, F_3,18_ = 7.1, *p* = 0.002; *Fisher’s LSD post-hoc test: Rot. + NAC. vs. control, *p* = 0.001. Proton Leak ANOVA, F_3,18_ = 4.3, *p* = 0.01; *Fisher’s LSD post-hoc test: Rot. + NAC vs. control, *p* < 0.004. Maximal Respiration ANOVA, F_3,18_ = 7.7, *p* = 0.001; *Fisher’s LSD post-hoc test: Rot. + NAC vs. control and vs. NAC, *p* < 0.004. Spare Respiratory Capacity ANOVA, F_3,18_ = 5.6, *p* = 0.006; *Fisher’s LSD post-hoc test: Rot. + NAC vs. control and vs. NAC, *p* < 0.02. ATP-Linked OCR ANOVA, F_3,18_ = 6.05, *p* = 0.005; *Fisher’s LSD post-hoc test: Rot. + NAC vs. control, *p* < 0.02. **Mn-Tbap: K.** Added for the last 24 h of the exposure to rotenone for 72 h. One sample exceeding mean ± 2SD was omitted. Basal OCR ANOVA, F_3,13_ = 24.5, *p* = 0.00001; *Fisher’s LSD post-hoc test: Mn-Tbap vs. control, *p* < 0.01; Rot. + Mn-Tbap vs. Rot., *p* < 0.01. Proton Leak ANOVA, F_3,14_ = 18.8, *p* = 0.00003; *Fisher’s LSD post-hoc test: Mn-Tbap and Rot. + Mn-Tbap vs. control and vs. Rot., *p* < 0.0002. **L.** Added for the last 48 h of the exposure to rotenone for 96 h. Basal OCR ANOVA, F_3,15_ = 9.3, *p* = 0.001; *Fisher’s LSD post-hoc test: Mn-Tbap Rot. + Mn-Tbap vs. control, *p* < 0.004. Proton Leak ANOVA, F_3,16_ = 4.7, *p* = 0.01; *Fisher’s LSD post-hoc test: Rot. + Mn-Tbap and Mn-Tbap vs. Rot. and vs. control, *p* < 0.02. Maximal Respiration ANOVA, F_3,16_ = 10.1, *p* = 0.0005; *Fisher’s LSD post-hoc test: Mn-Tbap and Rot. + Mn-Tbap vs. control *p* < 0.004. Spare Respiratory Capacity ANOVA, F_3,16_ = 6.1, *p* = 0.005; *Fisher’s LSD post-hoc test: Mn-Tbap and Rot. + Mn-Tbap vs. control, *p* < 0.02. Non-mitochondrial Respiration ANOVA, F_3, 14_ = 3.9, *p* = 0.03; *Fisher’s LSD post-hoc test: Mn-Tbap vs. all, *p* < 0.05. ATP-Linked OCR ANOVA, F_3,16_ = 9.4, *p* = 0.0007; *Fisher’s LSD post-hoc test: Rot. + Mn-Tbap and Mn-Tbap vs. control, *p* < 0.005.

Results measured as OD_540 nm_ are expressed in percent of control. Depicted are means of three experiments, each in triplicate ± S.E.M. Control value was: 7.2 ± 2.2 OD_540 nm_. Rot. = rotenone; Li = lithium; Tre. = trehalose; Rap. = rapamycin; Res. = resveratrol; NAC = N-acetylcysteine; Mn = Mn-Tbap. ^з^Rot. for 96 h vs. control, *p* > 0.05—as previously described [23] and confirmed now. **A.** Rapamycin effects, Two-way ANOVA: Treatment—F_3.16_ = 23.8, p = 0.000001; duration—F_1.16_ = 89.9, *p* = 0.000001; TreatmentXDuration interaction—F_3,16_ = 84.9, *p* = 0.000001; *Fisher’s LSD post-hoc test: Rot. for 72 h + Rap. last 24 h vs. 72 h of Rot., *p* < 0.04; 48 h of Rap. vs. all, *p* < 0.0006; Rot. for 96 h. + Rap. last 48 h vs. all, *p* < 0.05. **B.** Resveratrol effects, Two-way ANOVA: Treatment—F_3,16_ = 197.1, *p* = 0.000001; duration—F_1.16_ = 23.3, *p* = 0.0003; TreatmentXDuration interaction—F_3,16_ = 84.89, *p* = 0.000001; *Fisher’s LSD post-hoc test: 24 and 48 h of Res. vs. all, *p* < 0.05; Rot. for 72 h + Res. last 24 h vs. all, *p* < 0.05. One sample exceeding mean ± 2SD was omitted. **C.** NAC effects, Two-way ANOVA: Treatment—N.S.; duration—F_1,18_ = 5.5, *p* = 0.03; TreatmentXDuration interaction—F_3,18_ = 6.3, *p* = 0.004; *Fisher’s LSD post-hoc test: 24 h of NAC vs. all, *p* < 0.05; Rot. for 96 h. + NAC. last 48 h vs. Rot. for 96 h., *p* < 0.05. One sample exceeding mean ± 2SD was omitted. **D.** Mn-Tbap effects, Two-way ANOVA: Treatment—F_3,16_ = 6.7, *p* = 0.001; duration—N.S.; TreatmentXDuration interaction—F_3,16_ = 11.855, *p* = 0.00024; *Fisher’s LSD post-hoc test: 48 h of Mn-Tbap vs. 24 h of Mn-Tbap and vs. control, *p* < 0.02; Rot. for 96 h. + Mn-Tbap last 48 h vs. all, *p* < 0.05.

## 4. Discussion

Mitochondrial dysfunction characterizes multiple seemingly unrelated disorders, including mitochondrial diseases, diabetes, neurodegenerative disorders, cancer, cardiovascular disease, psychiatric disorders, chronic fatigue syndrome, epilepsy, migraine headaches, strokes, neuropathic pain, chronic fatigue syndrome, fibromyalgia, retinitis pigmentosa, hepatitis C, primary biliary cirrhosis and aging and senescence [41]. The degree of the dysfunction differs significantly among them. In our recent study [23], we chose exposure to 10 pM rotenone for 72/96 h to induce mild mitochondrial damage mimicking, among others, the situation in BPD [6,42]. The central aim of the present study was to investigate whether autophagy enhancers/ROS scavengers can alleviate the changes induced by this low-dose rotenone.

At therapeutically relevant concentrations, lithium salts (Li), the prototype treatment of BPD [43], facilitate the clearance of known autophagy substrates [44], such as mutated huntingtin and α-synuclein, and induces clearance of protease-resistant prion proteins [45]. Li’s autophagy-inducing properties were hypothesized to contribute to its protective effects in ALS [46], and its use in combination with rapamycin has been proposed as a rational therapy in Huntington’s disease models [47]. The elevated LC3-II protein levels following 24 h of Li treatment corroborate previous reports of Li-induced enhanced autophagy [48].

When Li was added for the last 24/48 h of the exposure to rotenone for 72/96 h, respectively, the levels of the OCR parameters became non-significantly different neither from those of rotenone only, nor from control levels. It can be, cautiously, interpreted that Li partially reversed rotenone’s effect. These effects may have been mediated via annulment of rotenone’s effect on complex I activity [23,35,49]. Unlike others [35,49], we did not find that lithium by itself increases CoI protein levels and activity. However, the significant increase in cell viability following exposure to rotenone for 96 h with lithium added for the last 48 h is compatible with lithium inducing neurogenesis [50,51,52], and could explain its effect of reversing rotenone’s effect on OCR parameters. Li’s effect of counteracting rotenone’s effects on OCR parameters both following exposure to rotenone for 72 h and 96 h is, apparently, mediated by different mechanisms. Namely, Li’s effect of normalizing mitochondrial respiration in cells exposed to rotenone for 72 h coincided with the effect of the drug to counteract the increase in complex I activity caused by rotenone. As for Li’s effect of normalizing mitochondrial respiration in cells exposed to rotenone for 96 h (down regulation), it coincided with normalizing LC3-II protein levels, and, accordingly, increasing cell viability with a mild change in complex I activity.

Trehalose, a natural disaccharide found in several organisms, is an important autophagy modulator suggested as a treatment for neurodegenerative diseases in which autophagy has been shown to play a role [33,53]. In animal models, trehalose was found to stimulate autophagy through the adenosine monophosphate-activated protein kinase (AMPK) [54]; however, its role in autophagy is still controversial [55]. Differently from other groups [33,54], we used 50 mM trehalose (for 24/48 h) rather than 100 mM because in combination with rotenone it was too toxic for the SH-SY5Y cells. Our results indicated a significant increase in non-mitochondrial respiration following 24 h of exposure. But, when trehalose was added for the last 24 h of the 72 h of rotenone, it induced reduction in cell viability (assayed by the MTT assay, which is based on mitochondrial activity), and a parallel reduction in CoIV protein levels, interpretable as a reduction in the number of mitochondria [56]. Employing 100 mM trehalose for 48 h resulted in an elevation in LC3-II protein levels [33]. The lack of such an effect in the present study is probably due to the use of 50 mM, rather than 100 mM.

It is of interest to note that Li added for the last 24 h of the 72 h of exposure to rotenone affected rotenone-induced increased respiration in a similar manner to trehalose. Namely, each of the drugs annulled rotenone’s effect, reflected in normalizing the levels to those of the control. However, unlike Li, trehalose did not affect the reduction in OCR parameters following exposure to rotenone for 96 h.

Rapamycin, a specific inhibitor of mTOR [34], was shown to increase mitophagy in SH-SY5Y cells (1 mM for 48 h) [57], as well as in in vivo models [58]. Corroborating the report of rapamycin’s effect in an animal model of ischemic stroke [58], when added for the last 24 h of the exposure to rotenone for 72 h, rapamycin had a strong impact. It significantly increased mitochondrial membrane potential (MMP) and significantly reversed rotenone’s effects on OCR parameters. As in the case of Li and trehalose, rapamycin added for the last 48 h of the exposure to rotenone for 96 h presented a different profile from that discussed above. The drug did not affect rotenone-induced toxicity on respiration and even significantly decreased cell viability measured based on mitochondrial activity (MTT assay), but it did elevate ATP levels. The latter is compatible with findings that rapamycin, by inhibiting mTOR, decreases neurogenesis [58], neural stem cell proliferation [59] and mitochondrial respiration [60]. It is, therefore, conceivable that the effect on ATP levels was not mediated via the drug’s effect on mitochondrial respiration enhancement. Rather, as demonstrated by Li and his colleagues [58], it might be attributed to a rapamycin-induced decrease in mitochondrial respiration, which might have caused elevated glycolysis, thereby resulting in increased ATP levels. This supposition corroborates with Ramanathan et al.’s report of rapamycin-induced increased aerobic glycolysis in leukemic cells [60]. Alternatively, increased ATP levels induced by rapamycin might reflect inhibition of ATP usage, e.g., decrease in protein synthesis, a major energy-consuming process, which may account for its ATP-saving effect [61].

Resveratrol, a Sirt1 stimulator belonging to the class III histone deacetylases family [62], plays a role in autophagy regulation through mTOR inhibition [38]. It is also known as a ROS scavenger [63,64]. It has been reported that resveratrol may exert both antioxidant and pro-oxidant effects in mammalian cells depending on its concentration [65]. We demonstrate that resveratrol’s effects are duration-dependent. Namely, exposure for 24 h decreased cell viability (based on the MTT assay), corroborating with the decrease in ATP levels and with others’ reports that resveratrol decreases neurogenesis [66,67], possibly via mTOR inhibition [66]. On the other hand, exposure to resveratrol for 48 h resulted in an increase in apoptosis, mitochondrial mass, ATP levels, and CoIII protein levels, reminiscent of cytotoxic effects of this polyphenol [68,69]. Resveratrol added for the last 24 h of the exposure to rotenone for 72 h reversed the elevated respiration. As mentioned above for rapamycin’s effect, this downregulation of ETC activity, accompanied by elevated ATP levels, could be attributed to either increased glycolytic flux [70] or inhibition of ATP usage.

Resveratrol added for the last 48 h of the exposure to rotenone for 96 h did not affect a rotenone-induced reduction in OCR levels. As in the case of rapamycin, it significantly reduced OCR parameters and cell viability measured based on mitochondrial activity (MTT assay). These results both corroborate [71,72,73] and oppose [74,75] other reports related to resveratrol’s effects on the mitochondria in different cell cultures. The drug’s effect of elevating mitochondrial mass may, possibly, reflect a compensatory effect that, nevertheless, was not capable of restoring mitochondrial respiration.

N-acetylcysteine (NAC) affects multiple pathophysiological targets, including neurotransmitters (glutamate and dopamine) and the intrinsic antioxidant glutathione (GSH). GSH, the main antioxidant in the brain, scavenges ROS (and nitrous oxide). These effects decrease cellular damage [36]. In addition, administration of NAC has been shown to promote neurogenesis both directly, by increasing the levels of neuroprotective proteins, such as brain-derived neurotrophic factor (BDNF), and indirectly, by reducing apoptosis through an increase in antiapoptotic proteins, such as Bcl-2 [76]. All these potentially therapeutic actions made this compound a promising drug candidate for neuropsychiatric disorders [76,77]. In previous studies, using at least 1 mM NAC for 72 h [78], increased ATP levels along with downregulation of autophagy and of LC3-II protein levels [79] were obtained. The effect of NAC on apoptosis is, apparently, exposure duration-, dose-, and cell type-dependent. Under the conditions of the present study, exposure for 48 h increased apoptosis, corroborating with some reports [80,81,82], but in contrary with others [83]. NAC added for the last 24 h of the exposure to rotenone for 72 h reversed the rotenone-induced increase in mitochondrial respiration, possibly via normalizing CoI activity to control levels. Interestingly, NAC added for the last 48 h of the exposure to rotenone for 96 h increased cell viability, an effect compatible with other reports of NAC’s effect to promote neurogenesis [84]. Under these conditions, NAC also reversed the rotenone-induced reduction in ATP levels, but it did not correct the reduction in mitochondrial OCR parameters or in CoI activity. Hence, the mechanism of the correction of ATP levels could be via anaerobic respiration, as discussed for the results following rapamycin and resveratrol treatment.

Mn-Tbap is a cell-permeable superoxide dismutase mimetic and a potent inhibitor of oxidation [37]. Exposure to Mn-Tbap for 24 h caused a significant temporary decrease in mitochondrial mass. The effect disappeared following exposure for an additional 24 h (altogether 48 h) to the same dose. Other groups also reported a decrease in mitochondrial mass in response to Mn-Tbap, although when using a 100-fold higher concentration than ours, and with the drug given in combination with stress induction [85,86]. Forty-eight h of exposure to Mn-Tbap did reduce basal- and ATP-linked OCR along with a significant increase in non-mitochondrial respiration. The reduction might reflect an escalation of the reduction in mitochondrial mass observed following 24 h of exposure.

Mn-Tbap reversed the rotenone-induced effect on OCR parameters when added for the last 24 h of the exposure to rotenone for 72 h. As in the case of the other drugs, this might be attributed to the non-significant trend of reduction in CoI activity. When the drug was added for the last 48 h of the exposure to rotenone for 96 h, similarly to rapamycin, resveratrol and NAC, it did not reverse the rotenone-induced reduction in OCR parameters. This implies that the mechanism by which these four drugs increase ATP levels in this paradigm is not via enhancing mitochondrial respiration. Neither Mn-Tbap nor lithium added for the last 24 h of the exposure to rotenone for 72 h counteracted rotenone’s effect to increase LC3-II protein levels. In the case of lithium, the reason for this is, conceivably, due to the drug’s effect by itself to elevate these levels, i.e., a ceiling effect. On the other hand, in the case of Mn-Tbap, it coincides with the observation that when added by itself, the drug did not affect LC3-II protein levels.

Resveratrol, rapamycin, and Mn-Tbap added for 48 h had the same effect on OCR parameters as that of rotenone’s following exposure for 96 h. Hence, it is not surprising that the three drugs did not counteract rotenone’s effect. This corroborates with the drugs’ decreasing effect on cell viability observed under the same conditions. This mirrors the results with lithium which, when added for the last 48 h of the exposure to rotenone for 96 h increased cell viability and counteracted rotenone’s reducing effects of OCR parameters. These lithium’s effects are reminiscent of the drug’s effect to stimulate the proliferation of hippocampal progenitor cells in vitro [87], although data regarding lithium’s ability to increase neuronal differentiation and survival are equivocal [52,88,89].

To sum up, using our previously designed rotenone’s regime [23] we replicated the upregulation of most mitochondrial respiration parameters and non-mitochondrial respiration following 72 h of exposure and downregulation of all of them, following 96 h. The same directions of effects were observed for the autophagy marker LC3-II protein levels and CoI activity. Since our rotenone regime does not affect ROS levels, reflecting mild mitochondrial distress, it might have been expected that the ROS scavengers will not affect rotenone-induced effects. Intriguingly, all autophagy enhancers and ROS scavengers studied exhibited the same counteracting effects on rotenone-induced effects following 72 h, and only lithium counteracted rotenone’s effect on mitochondrial respiration following 96 h of exposure. Therefore, the mechanism mediating the drugs’ effects remains to be further investigated. Nevertheless, the present results raise the possibility that the drugs studied may be considered as an add-on to lithium treatment, particularly the GRAS compounds.

## 5. Limitations

We used the neuroblastoma cell model, SH-SY5Y. Hence, despite being derived from neuronal tissue and exhibiting neuronal-like characteristics, results obtained with these cells may not be unequivocally extrapolated to neuronal in vivo settings.

The study’s focus is mitochondrial function. Nevertheless, cell viability was assessed using a methodology based on mitochondrial activity (the MTT assay). This may have caused an intrinsic erroneousness.

Lastly, our results concerning the effect of the various drugs on autophagy are based on protein levels of the process’ markers that do not monitor the flux and do not discriminate between autophagy and mitophagy. Future studies of additional parameters [90] are required to further elaborate on this issue.

## Figures and Tables

**Table 1:** A qualitative summary of the effects of ROS scavengers/autophagy enhancers, per se, on mitochondrial parameters.

Drug Parameters		vs. Control											
		Lithium 24 hrs	Trehalose 24 hrs	Rapamycin 24 hrs	Resveratrol 24 hrs	NAC 24 hrs	Mn-Tbap 24 hrs	Lithium 48 hrs	Trehalose 48 hrs	Rapamycin 48 hrs	Resveratrol 48 hrs	NAC 48 hrs	Mn-Tbap 48 hrs
**Cell viability**	**MTT assay**	**↔**	**↔**	**↔**	**↓**	**↔**	**↔**	**↔**	**↔**	**↔**	**↔**	**↔**	**↑**
	**ApoLive-Glo multiplex assay**	**↔**	**↔**	**↔**	**↔**	**↔**	**↔**	**↔**	**↔**	**↔**	**↑**	**↑**	**↔**
**Mito. characteristics**	**Mito. mass**	**↔**	**↔**	**↔**	**↔**	**↔**	**↓**	**↔**	**↔**	**↔**	**↑**	**↔**	**↔**
	**MMP (ΔψM)**	**↔**	**↔**	**↔**	**↔**	**↔**	**↔**	**↔**	**↔**	**↔**	**↔**	**↔**	**↔**
	**ROS**	**↔**	**↔**	**↔**	**↔**	**↔**	**↔**	**↔**	**↔**	**↔**	**↔**	**↔**	**↔**
**Mito. respiration**	**Basal OCR**	**↔**	**↔**	**↔**	**↔**	**↔**	**↓**	**↓**	**↔**	**↔**	**↓**	**↔**	**↓**
	**Proton leak**	**↔**	**↔**	**↔**	**↔**	**↔**	**↓**	**↔**	**↔**	**↓**	**↔**	**↔**	**↓**
	**Maximal respiration**	**↔**	**↔**	**↑**	**↔**	**↔**	**↔**	**↔**	**↔**	**↔**	**↓**	**↔**	**↓**
	**Spare respiratory capacity**	**↔**	**↔**	**↑**	**↔**	**↔**	**↔**	**↔**	**↔**	**↔**	**↓**	**↔**	**↓**
	**ATP-linked OCR**	**↔**	**↔**	**↔**	**↔**	**↔**	**↔**	**↔**	**↔**	**↓**	**↓**	**↔**	**↓**
	**Non mito. respiration**	**↔**	**↑**	**↔**	**↔**	**↔**	**↔**	**↔**	**↔**	**↔**	**↔**	**↔**	**↑**
**ATP**	**Levels**	**↔**	**↑**	**↔**	**↓**	**↑**	**↔**	**↔**	**↑**	**↑**	**↑**	**↔**	**↑**
**Autophagy markers**	**LC3-II**	**↑**	**↑**	**↔**	**↔**	**↔**	**↔**	**↔**	**↔**	**↔**	**↔**	**↔**	**↔**
	**p62**	**↔**	**↔**	**↔**	**↔**	**↔**	**↔**	**↔**	**↔**	**↔**	**↔**	**↔**	**↔**
**Complex I**	**Activity**	**↔**	**↔**	**↔**	**↔**	**↔**	**↔**	**↔**	**↔**	**↔**	**↔**	**↔**	**↔**
	**Protein levels**	**↔**	**↔**	**↔**	**↔**	**↔**	**↔**	**↔**	**↔**	**↔**	**↔**	**↔**	**↔**
**Mito. Complexes II-V**	**CoII protein levels**	**↔**	**↔**	**↔**	**↔**	**↔**	**↔**	**↔**	**↔**	**↔**	**↔**	**↔**	**↔**
	**CoIII protein levels**	**↔**	**↔**	**↔**	**↔**	**↔**	**↔**	**↔**	**↔**	**↔**	**↑**	**↔**	**↔**
	**CoIV protein levels**	**↔**	**↑**	**↔**	**↔**	**↔**	**↑**	**↔**	**↔**	**↔**	**↔**	**↔**	**↔**
	**CoV protein levels**	**↔**	**↔**	**↔**	**↔**	**↔**	**↔**	**↔**	**↔**	**↔**	**↔**	**↔**	**↔**

↔ = No change; ↑ = Increase; ↓ = Decrease; Mito. = mitochondrial; Rot. = rotenone; Li = lithium; Tre. = trehalose; Rap. = rapamycin; Res. = resveratrol; NAC = N-acetylcysteine; Mn. = Mn-Tbap.

**Table 2:** A qualitative summary of the interaction between the effects of rotenone and ROS scavengers/ autophagy enhancers.

Drug Parameters		vs. Control													
		Rot. for 72 hrs	Rot.+Li. Last 24 hrs	Rot.+Tre. Last 24 hrs	Rot.+Rap. Last 24 hrs	Rot.+Res. Last 24 hrs	Rot.+NAC Last 24 hrs	Rot.+Mn. Last 24 hrs	Rot. for 96 hrs	Rot.+Li. Last 48 hrs	Rot.+Tre. Last 48 hrs	Rot.+Rap. Last 48 hrs	Rot.+Res. Last 48 hrs	Rot.+NAC Last 48 hrs	Rot.+Mn. Last 48 hrs
**Cell viability**	**MTT assay**	**↔**	**↔**	**↓**	**↔**	**↓**	**↔**	**↓**	**↔**	**↑**	**↔**	**↓**	**↓**	**↑**	**↓**
	**ApoLive-Glo multiplex assay**	**↔**	**↔**	**↔**	**↔**	**↔**	**↔**	**↔**	**↔**	**↔**	**↔**	**↔**	**↔**	**↔**	**↔**
**Mito. Characteristics**	**Mito. mass**	**↔**	**↔**	**↔**	**↔**	**↔**	**↔**	**↓**	**↔**	**↔**	**↔**	**↔**	**↑**	**↔**	**↔**
	**MMP (ΔψM)**	**↔**	**↔**	**↔**	**↑**	**↔**	**↔**	**↔**	**↔**	**↔**	**↔**	**↔**	**↔**	**↔**	**↔**
	**ROS**	**↔**	**↔**	**↔**	**↔**	**↔**	**↔**	**↔**	**↔**	**↔**	**↔**	**↔**	**↔**	**↔**	**↔**
**Mito. respiration**	**Basal OCR**	**↑**	**↔**	**↔**	**↓**	**↓**	**↓**	**↓**	**↓**	**↔**	**↓**	**↓**	**↓**	**↓**	**↓**
	**Proton leak**	**↑** *	**↔**	**↔**	**↓**	**↓**	**↓**	**↓**	**↓**	**↔**	**↓**	**↓**	**↓**	**↓**	**↓**
	**Maximal respiration**	**↔**	**↔**	**↔**	**↓**	**↓**	**↔**	**↔**	**↓**	**↔**	**↓**	**↓**	**↓**	**↓**	**↓**
	**Spare respiratory capacity**	**↔**	**↔**	**↔**	**↓**	**↔**	**↔**	**↔**	**↓**	**↔**	**↓**	**↓**	**↓**	**↓**	**↓**
	**ATP- linked OCR**	**↑**	**↑**	**↔**	**↓**	**↓**	**↓**	**↔**	**↓**	**↔**	**↓**	**↓**	**↓**	**↓**	**↓**
	**Non mito. respiration**	**↑**	**↑**	**↑**	**↔**	**↔**	**↔**	**↔**	**↔**	**↔**	**↔**	**↔**	**↔**	**↔**	**↔**
**ATP**	**Levels**	**↔**	**↔**	**↔**	**↓**	**↑**	**↔**	**↔**	**↓**	**↔**	**↔**	**↑**	**↔**	**↑**	**↑**
**autophagy markers**	**LC3-II**	**↑**	**↑**	**↔**	**↔**	**↔**	**↔**	**↑**	**↓**	**↔**	**↔**	**↔**	**↔**	**↔**	**↔**
	**p62**	**↔**	**↔**	**↔**	**↔**	**↔**	**↔**	**↔**	**↔**	**↔**	**↔**	**↔**	**↔**	**↔**	**↔**
**Complex I**	**Activity**	**↑**	**↔**	**↔**	**↔**	**↔**	**↔**	**↔**	**↓**	**↔**	**↔**	**↔**	**↔**	**↔**	**↔**
	**Protein levels**	**↑**	**↔**	**↔**	**↓**	**↔**	**↔**	**↑**	**↔**	**↔**	**↔**	**↔**	**↔**	**↔**	**↔**
**Mito. Complexes II-V**	**CoII protein levels**	**↔**	**↔**	**↔**	**↔**	**↔**	**↔**	**↔**	**↔**	**↔**	**↔**	**↔**	**↔**	**↔**	**↔**
	**CoIII protein levels**	**↔**	**↔**	**↔**	**↔**	**↑**	**↔**	**↔**	**↔**	**↔**	**↔**	**↔**	**↔**	**↔**	**↔**
	**CoIV protein levels**	**↑**	**↔**	**↔**	**↔**	**↔**	**↔**	**↑**	**↑**	**↔**	**↑**	**↔**	**↔**	**↔**	**↔**
	**CoV protein levels**	**↔**	**↔**	**↔**	**↔**	**↔**	**↔**	**↔**	**↔**	**↔**	**↔**	**↔**	**↔**	**↔**	**↔**

* As previously described [23]. ↔ = No change; ↑ = Increase; ↓ = Decrease; Mito. = mitochondrial; Rot. = rotenone; Li = lithium; Tre. = trehalose; Rap. = rapamycin; Res. = resveratrol; NAC=N-acetylcysteine; Mn. = Mn-Tbap.

## Data Availability

All data are available in our lab’s files and can be obtained upon request.

## Supplementary Materials

The following are available online at https://www.mdpi.com/article/10.3390/ijms22115753/s1.
