# Supplementary material for: Do Autophagy Enhancers/ROS Scavengers Alleviate Consequences of Mild Mitochondrial Dysfunction Induced in Neuronal-Derived Cells?
Source: Int J Mol Sci. 2021 May 27;22(11):5753. doi: 10.3390/ijms22115753 (PMC8197898; doi:10.3390/ijms22115753)
Supplement: Supplementary file 1 [file ijms-22-05753-s001.zip › ijms-1178417-supplementary.pdf]

Damri *et al - Supplementary material*

A supplementary figure

A.

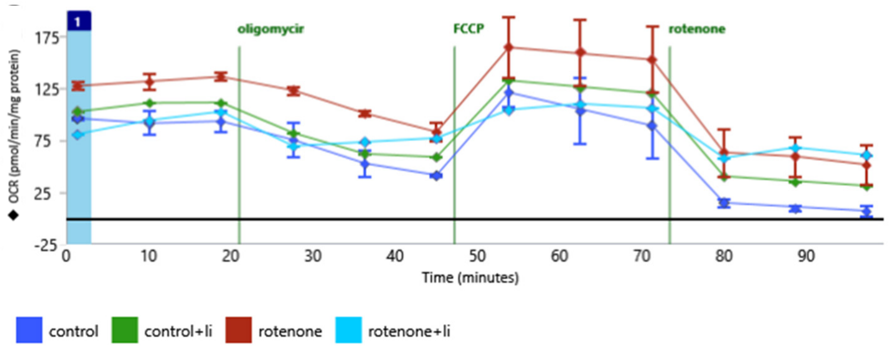

B.

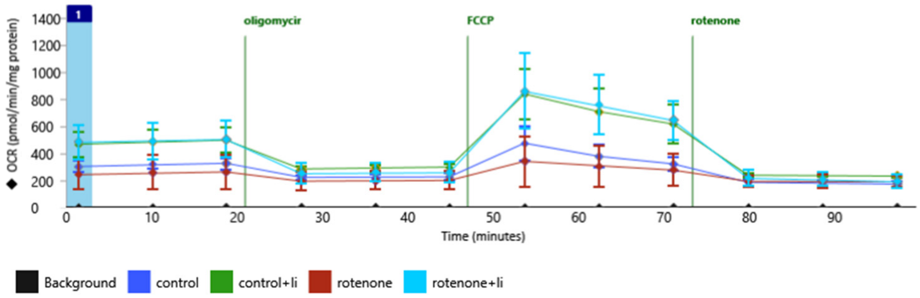

Supplementary figure S1: Representative graphs of the effect of 1 mM of lithium for 24 (A) and 48 (B) hrs either by itself or for the last 24 (A) or 48 (B) hrs of the exposure to 10 pM rotenone for 72 (A) and 96 (B) hrs on mitochondrial respiration parameters.
